# Prevalence of temporomandibular disorders and their association with oral behaviors, anxiety, and depression among medical and dental students in Brazil—a cross-sectional study

**DOI:** 10.22514/jofph.2026.008

**Published:** 2026-01-12

**Authors:** Mayana Cristina Silva Dantas, Arthur Nascimento Queiroz, Camilla Silva da Costa Muniz Franco, Anna Luiza Delmondes de Lima, Rodrigo Antonio de Medeiros, Mateus Veppo dos Santos

**Affiliations:** ^1^Department of Dentistry, School of Health Sciences, University of Brasília (UnB), 70.910-900 Brasilia, Brazil; ^2^Department of Dentistry, Euro-American University Center, 70.200-001 Brasilia, Brazil

**Keywords:** Students, dental, Students, medical, Temporomandibular joint disorders, Anxiety, Depression

## Abstract

**Background**: Temporomandibular disorders (TMD) are a group of musculoskeletal conditions that frequently affect the masticatory muscles and temporomandibular joints and often result in pain, dysfunction, and decreased quality of life. University students, particularly those in health-related fields, may be especially vulnerable to TMD owing to their elevated stress levels and the presence of oral parafunctional behaviors. Psychosocial factors such as anxiety and depression are recognized as important contributors to the onset and exacerbation of TMD symptoms. **Methods**: This cross-sectional study included 182 undergraduate students enrolled in medical and dental programmes at a private university in Brazil. The participants completed an online self-report questionnaire that included the Diagnostic Criteria for Temporomandibular Disorders (DC/TMD) Symptom Questionnaire, Oral Behavior Checklist (OBC), Generalized Anxiety Disorder Scale (GAD-7), and Patient Health Questionnaire (PHQ-9). Associations among TMD symptoms, oral behaviors, anxiety, and depression were analyzed using the chi-square and Fisher’s exact tests (α = 0.05). **Results**: A total of 78.6% of the participants reported TMD symptoms, with 38.5% experiencing both pain-related and joint-related symptoms. Oral behaviors were reported by 98.9% of the students, while symptoms of anxiety and depression were present in 74.7% and 65.4% of the sample, respectively. Significant associations were found between painful/joint TMD and higher levels of oral behaviors (*p * < 0.001), severe anxiety (*p* = 0.015), and moderately severe to severe depression (*p* = 0.016). Oral behaviors were more frequent in students in later semesters (*p* = 0.006) and were associated with anxiety (*p * < 0.001) and depression (*p * < 0.001). A strong correlation was observed between anxiety and depression (*p * < 0.001). **Conclusions**: This study confirmed a high prevalence of temporomandibular disorder (TMD) symptoms among medical and dental students. Pain-related and joint-related TMD symptoms were significantly associated with high-frequency oral behaviors, as well as with moderate to severe levels of anxiety and depression.

## 1. Introduction

Temporomandibular disorders (TMD) are a group of musculoskeletal and neuromuscular conditions that affect the temporomandibular joints (TMJ), masticatory muscles, and associated structures. Clinical features often include pain, joint sounds, and restricted mandibular movement, leading to significant functional impairment and reduced quality of life [1, 2]. TMD is one of the most common non-dental causes of orofacial pain and is a multifactorial condition [3, 4].

Although early research emphasized occlusal discrepancies as a primary etiological factor, recent evidence highlights the influence of psychological and behavioral aspects, including anxiety, depression, stress, and oral parafunctional behaviors such as bruxism [5, 6, 7]. These factors are particularly relevant in university settings, where students, especially those in health-related fields, are exposed to academic stress, clinical responsibilities, and performance pressure [8, 9]. Such stressors can exacerbate muscle tension and contribute to oral behaviors, such as clenching or grinding, which in turn may aggravate TMD symptoms [10, 11].

Considered a significant public health issue, temporomandibular disorder (TMD) affects approximately 5% to 12% of the global population and is the second most common musculoskeletal condition associated with pain and disability [12]. In Brazil, it is estimated that around 37% of the population presents at least one symptom of TMD, although only 15% of these cases require treatment. The condition is more prevalent in women, particularly within the age range of 19 to 45 years [13].

Psychological factors such as depression, anxiety, and catastrophizing play a substantial role not only in the onset but also in the persistence of TMD, negatively impacting treatment outcomes [14]. The biopsychosocial model has emphasized how psychosocial factors—such as stress and anxiety—can contribute to the development of TMD, including the influence on parafunctional habits. Patients affected by these psychosocial factors often exhibit higher levels of anxiety, depression, stress, and pain intensity [15].

Several studies have reported a high prevalence of TMD symptoms among university students, although the rates vary depending on the tools used and the population studied [7, 16, 17]. However, few studies have simultaneously assessed the interrelationship between TMD symptoms, oral behaviors, and psychological distress, such as anxiety and depression, in this population.

Therefore, the present study aimed to evaluate the prevalence of TMD symptoms among undergraduate medical and dental students and to investigate the associations between TMD, oral behaviors, and symptoms of anxiety and depression. It was hypothesized that a high prevalence of TMD would be observed in this population, with significant associations between TMD symptoms, oral parafunctional behaviors, and psychological distress.

## 2. Materials and methods

### 2.1 Study design and ethical approval

A cross-sectional study was conducted at Euro-American University Center, including campuses located in Asa Sul and Águas Claras (Brasília, Brazil). The study protocol was reviewed and approved by the university’s Institutional Review Board (approval number: 7.399.261), and all participants provided informed consent electronically before participation. Students were invited to participate in the research voluntarily, without any form of coercion due to their academic status. Those who agreed to participate in the study were duly informed that their participation was entirely voluntary and that they could withdraw at any time without any penalty.

### 2.2 Sample size and participants

The sample size was calculated using OpenEpi version 2.3, assuming a 15% prevalence of TMD in the general population, 95% confidence interval, 5% margin of error, and a design effect of 1.0. The minimum required sample size was 172 students. From a total population of approximately 1400 students enrolled in undergraduate medical and dental programs (550 in medicine and 850 in dentistry), a simple random sample of 182 students was selected. The inclusion criteria were as follows: (1) being at least 18 years old; (2) actively enrolled in the medical or dental program; and (3) agreement to participate voluntarily and to complete the full questionnaire. Students younger than 18 years were excluded.

### 2.3 Data collection

The data collection was conducted between February and March 2025. The participants completed a self-administered online questionnaire composed of five sections: sociodemographic data, TMD symptoms, anxiety symptoms, depressive symptoms, and oral behaviors.

TMD symptoms were assessed using the Diagnostic Criteria for Temporomandibular Disorders (DC/TMD) Symptom Questionnaire, which was validated in Brazilian Portuguese. Based on responses referring to the past 30 days, participants were categorized into four groups: no TMD, joint-related TMD, pain-related TMD, and combined TMD. Categorization was performed according to the diagnostic flow outlined in the DC/TMD guidelines.

Anxiety symptoms were measured using the Generalized Anxiety Disorder 7-item scale (GAD-7). Scores of 5, 10, and 15 were used as the cutoffs for mild, moderate, and severe anxiety, respectively.

Depressive symptoms were assessed using the Patient Health Questionnaire (PHQ-9). The total scores were classified as follows: 0–4 (none); 5–9 (mild); 10–14 (moderate); 15–19 (moderately severe); and 20–27 (severe).

Oral behavior was evaluated using the Oral Behavior Checklist (OBC). The total scores were grouped as follows: 0 (no oral behaviors), 1–24 (low frequency), and 25–84 (high frequency).

### 2.4 Statistical analysis

All data were analyzed using JAMOVI statistical software. Descriptive statistics were reported as absolute and relative frequencies. Associations between categorical variables were tested using the chi-squared test and Fisher’s exact test, as appropriate. A significance level of 5% (α = 0.05) was used for all the analyses.

## 3. Results

A total of 182 students, including 140 females (76.9%) and 42 males (23.1%), completed the survey. Of these, 66 (36.3%) were enrolled in the medical program and 116 (63.7%) were enrolled in the dental program. The mean age of the participants was 22 years. Regarding age groups, 30.2% were under 20 years, 63.2% were between 21 and 30 years, and 6.6% were over 30 years. Regarding academic level, 32.9% were in the early semesters (1st–4th), 31.4% were in the mid-stage (5th–7th), and 35.7% were in the final semesters (8th–12th).

Concerning TMD symptoms, 21.4% of students reported no symptoms, 17.5% had joint-related symptoms only, 22.6% had pain-related symptoms only, and 38.5% had both pain- and joint-related symptoms.

Regarding oral behaviors, 1.1% of the students reported no behaviors, 68.7% reported low-frequency behaviors, and 30.2% reported high-frequency behaviors. Anxiety symptoms were absent in 25.3% of the students; 35.7% had mild symptoms, 24.7% had moderate symptoms, and 14.3% had severe symptoms. Regarding depressive symptoms, 34.6% had no symptoms, 28.5% had mild symptoms, 18.7% had moderate symptoms, 11% had moderately severe symptoms, and 7.2% had severe symptoms.

Significant associations were found between TMD symptom categories and oral behavior frequency (*p * < 0.001), anxiety level (*p* = 0.015), and depression level (*p* = 0.016). Students with a high oral behavior frequency, severe anxiety, and moderately severe to severe depression were more likely to present with both pain-related and joint-related TMD symptoms (Table 1).

**Table 1.** Frequency (percentage) of students according to the association between temporomandibular disorder (TMD) symptoms and associated variables (Chi-square and Fisher’s exact tests, *p * < 0.05).

Variables		TMD Symptoms				*p*-value
		No TMD	Joint-related symptoms	Pain-related symptoms	Pain- and joint-related symptoms
Gender^†^						
	Female	28 (20.0%)	21 (15.0%)	36 (25.7%)	55 (39.3%)	0.126
	Male	11 (26.2%)	11 (26.2%)	5 (11.9%)	15 (35.7%)	
Age^‡^ (yr)						
	15–20	14 (25.5%)	8 (14.5%)	15 (27.3%)	18 (32.7%)	0.785
	21–30	22 (19.1%)	22 (19.1%)	23 (20.0%)	48 (41.7%)	
	>31	3 (25.0%)	2 (16.7%)	3 (25.0%)	4 (33.3%)	
Oral Behaviors^‡^						
	None	2 (100%)	0 (0%)	0 (0%)	0 (0%)	<0.001*
	Low	34 (27.2%)	25 (20.0%)	29 (23.2%)	37 (29.6%)	
	High	3 (5.5%)	7 (12.7%)	12 (21.8%)	33 (60.0%)	
Course^†^						
	Medicine	12 (18.2%)	7 (10.6%)	20 (30.3%)	27 (40.9%)	0.097
	Dentistry	27 (23.3%)	25 (21.6%)	21 (18.1%)	43 (37.1%)	
Course period^†^						
	1st–4th	15 (25.0%)	7 (11.7%)	15 (25.0%)	23 (38.3%)	0.355
	5th–7th	13 (22.8%)	15 (26.3%)	11 (19.3%)	18 (31.6%)	
	8th–12th	11 (16.9%)	10 (15.4%)	15 (23.1%)	29 (44.6%)	
Anxiety symptoms^‡^						
	None	14 (30.4%)	11 (23.9%)	7 (15.2%)	14 (30.4%)	0.015*
	Mild	18 (27.7%)	12 (18.5%)	17 (26.2%)	18 (27.7%)	
	Moderate	6 (13.3%)	6 (13.3%)	12 (26.7%)	21 (46.7%)	
	Severe	1 (3.8%)	3 (11.5%)	5 (19.2%)	17 (65.4%)	
Depression symptoms^‡^						
	None	23 (36.5%)	13 (20.6%)	8 (12.7%)	19 (30.2%)	0.016*
	Mild	8 (15.4%)	12 (23.1%)	16 (30.8%)	16 (30.8%)	
	Moderate	5 (14.7%)	4 (11.8%)	9 (26.5%)	16 (47.1%)	
	Moderately severe	2 (10.0%)	1 (5.0%)	5 (25.0%)	12 (60.0%)	
	Severe	1 (7.7%)	2 (15.4%)	3 (23.1%)	7 (53.8%)	

*p < 0.05, showing statistically significant difference. *^†^Chi-square Test.* *^‡^Fisher’s exact test.*

Further analysis revealed associations between oral behavior frequency and academic semesters (*p* = 0.006), anxiety symptoms (*p * < 0.001), and depressive symptoms (*p * < 0.001). High-frequency oral behaviors were most prevalent among students in the final semesters, as well as among those with moderate-to-severe anxiety and depression levels (Table 2).

**Table 2.** Frequency (percentage) of students according to the association between oral behavior frequency and associated variables (Fisher’s exact test, *p * < 0.05).

Variable		Oral Behaviors			*p*-value
		None	Low	High	
Gender					
	Female	1 (0.7%)	96 (68.6%)	43 (30.7%)	0.611
	Male	1 (2.4%)	29 (69.0%)	12 (28.6%)	
Age (yr)					
	15–20	0 (0%)	42 (76.4%)	13 (23.6%)	0.141
	21–30	2 (1.7%)	78 (67.8%)	35 (30.4%)	
	>31	0 (0%)	5 (41.7%)	7 (58.3%)	
Course					
	Medicine	1 (1.5%)	43 (65.2%)	22 (33.3%)	0.613
	Dentistry	1 (0.9%)	82 (70.7%)	33 (28.4%)	
Course period					
	1st–4th	2 (3.3%)	44 (73.3%)	14 (23.3%)	0.006*
	5th–7th	0 (0%)	45 (78.9%)	12 (21.1%)	
	8th–12th	0 (0%)	36 (55.4%)	29 (44.6%)	
Anxiety symptoms					
	None	2 (4.3%)	39 (84.8%)	5 (10.9%)	<0.001*
	Mild	0 (0%)	55 (84.6%)	10 (15.4%)	
	Moderate	0 (0%)	20 (44.4%)	25 (55.6%)	
	Severe	0 (0%)	11 (42.3%)	15 (57.7%)	
Depression symptoms					
	None	2 (3.2%)	53 (84.1%)	8 (12.7%)	<0.001*
	Mild	0 (0%)	41 (78.8%)	11 (21.2%)	
	Moderate	0 (0%)	21 (61.8%)	12 (35.3%)	
	Moderately severe	0 (0%)	6 (30.0%)	14 (70.0%)	
	Severe	0 (0%)	4 (30.8%)	9 (69.2%)	

*p < 0.05, showing statistically significant difference.

An additional association was found between anxiety and depression symptoms (*p * < 0.001), with severe anxiety being most frequently observed among students with severe depression (Table 3).

**Table 3.** Frequency (percentage) of students according to the association between anxiety symptoms and associated variables (Fisher’s exact test, *p * < 0.05).

Variables		Anxiety symptoms				*p*-value
		None	Mild	Moderate	Severe	
Gender						
	Female	31 (22.1%)	53 (37.9%)	34 (24.3%)	22 (15.7%)	0.278
	Male	15 (35.7%)	12 (28.6%)	11 (26.2%)	4 (9.5%)	
Age (yr)						
	15–20	14 (25.5%)	19 (34.5%)	15 (27.3%)	7 (12.7%)	0.998
	21–30	29 (25.2%)	42 (36.5%)	27 (23.5%)	17 (14.8%)	
	>31	3 (25.0%)	4 (33.3%)	3 (25.0%)	2 (16.7%)	
Course						
	Medicine	20 (30.3%)	24 (36.4%)	18 (27.3%)	4 (6.1%)	0.089
	Dentistry	26 (22.4%)	41 (35.3%)	27 (33.3%)	22 (19.0%)	
Course period						
	1st–4th	17 (28.3%)	22 (36.7%)	17 (28.3%)	4 (6.7%)	0.192
	5th–7th	13 (22.8%)	24 (42.1%)	9 (15.8%)	11 (19.3%)	
	8th–12th	16 (24.6%)	19 (29.2%)	19 (29.2%)	11 (16.9%)	
Depression symptoms						
	None	34 (54%)	24 (38.1%)	5 (7.9%)	0 (0%)	<0.001*
	Mild	9 (17.3%)	31 (59.6%)	10 (19.2%)	2 (3.8%)	
	Moderate	2 (5.9%)	6 (17.6%)	19 (55.9%)	7 (20.6%)	
	Moderately severe	0 (0%)	3 (15.0%)	11 (55%)	6 (30.0%)	
	Severe	1 (7.7%)	1 (7.7%)	0 (0%)	11 (84.6%)	

*p < 0.05, showing statistically significant difference.

No significant associations were observed between depressive symptoms and gender, age, academic program, or semester (Table 4).

**Table 4.** Frequency (percentage) of students according to the association between depression symptoms and associated variables (Fisher’s exact test, *p * < 0.05).

Variables		Depression symptoms					*p*-value
		None	Mild	Moderate	Moderately severe	Severe	
Gender							
	Female	46 (32.9%)	38 (27.1%)	27 (19.3%)	17 (12.1%)	12 (8.6%)	0.526
	Male	17 (40.5%)	14 (33.3%)	7 (16.7%)	3 (7.1%)	1 (2.4%)	
Age (yr)							
	15–20	19 (34.5%)	16 (29.1%)	12 (21.8%)	5 (9.1%)	3 (5.5%)	0.862
	21–30	41 (35.7%)	32 (27.8%)	21 (18.3%)	13 (11.3%)	8 (7.0%)	
	>31	3 (25.0%)	4 (33.3%)	1 (8.3%)	2 (16.7%)	2 (16.7%)	
Course							
	Medicine	24 (36.4%)	18 (27.3%)	12 (18.2%)	10 (15.2%)	2 (3.0%)	0.382
	Dentistry	39 (33.6%)	34 (29.3%)	22 (19.0%)	10 (8.6%)	11 (9.5%)	
Course period							
	1st–4th	23 (38.3%)	16 (26.7%)	12 (20.0%)	7 (11.7%)	2 (3.3%)	0.671
	5th–7th	20 (35.1%)	18 (31.6%)	11 (19.3%)	3 (5.3%)	5 (8.8%)	
	8th–12th	20 (30.8%)	18 (27.7%)	11 (16.9%)	10 (15.4%)	6 (9.2%)	

## 4. Discussion

The hypothesis of this study was supported as a high prevalence of TMD symptoms was observed among medical and dental students, along with significant associations with oral behaviors and psychological factors. In the present sample, 78.6% of the participants reported at least one TMD symptom, and 38.5% experienced both pain-related and joint-related manifestations. These rates are higher than those reported in some prior studies [2, 7, 18], but consistent with other studies that identified a similarly elevated prevalence among university students in health-related programs [19].

The high prevalence of TMD symptoms may be partially explained by the demanding academic environment and exposure to emotional stressors characteristic of medical and dental training [8, 9, 20]. In this context, the associations observed between TMD symptoms and severe anxiety, as well as moderately severe to severe depressive symptoms, align with earlier findings that highlight psychological distress as a contributing factor to orofacial pain [4, 5, 21, 22]. The interaction between psychosocial vulnerability and parafunctional activity may amplify musculoskeletal tension and lead to TMD onset or exacerbation of TMD.

Contrary to many studies reporting a female predominance in TMD prevalence [3, 7, 16], no significant association between sex and TMD symptoms was identified in this sample. This divergence may be attributed to similar academic stress levels across genders within this university context. Further investigations are required to clarify this observation in comparable populations. Additionally, the lack of statistical significance may be attributed to the sample size, given the limited participation of male students in the present study.

Oral behaviors were also significantly associated with TMD symptoms, supporting previous findings that parafunctional habits, especially clenching and grinding, are important contributors to TMD pathophysiology [7, 8, 10, 16]. Notably, behaviors were more prevalent among students in advanced semesters, possibly due to the increased clinical workload and responsibilities experienced during this phase of education [23].

Patients with temporomandibular disorders (TMD) consistently exhibit a higher frequency and intensity of non-functional oral behaviors compared to individuals without TMD, an association that becomes even more pronounced among those experiencing greater pain-related disability. These oral behaviors are not only linked to the presence of TMD but also correlate with increased pain severity, functional mandibular limitations, and psychological distress, particularly anxiety and depression. Mediation analyses suggest that such oral behaviors may act as a behavioral conduit between psychosocial factors (*e.g.*, anxiety) and the onset or persistence of TMD-related pain [24, 25].

Sleep bruxism was reported by nearly half of the participants (46.4%), a rate substantially higher than that observed in a Finnish student sample (17.9%). Waking oral behaviors, such as nonfunctional tooth contact, chewing gum use, and awake clenching, were also frequently reported, consistent with findings from other young populations [16].

The relationship between temporomandibular disorders (TMD), bruxism, and psychiatric factors—such as stress, anxiety, depression, and post-traumatic stress disorder (PTSD)—is complex, multifactorial, and bidirectional. Evidence suggests that individuals with PTSD exhibit a significantly higher prevalence of awake bruxism and orofacial pain, with anxiety serving as a key mediator linking TMD, bruxism, and psychiatric symptoms. This association is driven by psychosocial and neuroendocrine mechanisms through which emotional factors increase susceptibility to bruxism and temporomandibular pain/dysfunction. Therefore, clinical assessment should include a thorough evaluation of comorbid psychosocial factors. A multidisciplinary therapeutic approach that addresses both physical and psychological dimensions is essential to improving clinical outcomes in these conditions [26, 27, 28].

High-frequency oral behaviors were more common among students with moderate-to-severe anxiety and depression. This pattern reinforces the hypothesis that individuals experiencing emotional distress may manifest physical tension through masticatory muscle overuse, contributing to the maintenance or worsening of TMD [8, 16].

Although anxiety and depression were not associated with the academic semester, a strong correlation between the two was found, particularly among students with severe symptoms. This reinforces previous evidence of the interdependence of emotional disorders and their shared impact on pain perception and somatic symptom expression [21].

The present study presents several limitations. The sample size was limited to students from a single institution, which may have reduced the generalizability of our findings. Additionally, the symptoms of temporomandibular disorder (TMD) were self-reported, and no clinical examinations were conducted to confirm the diagnoses, potentially compromising the accuracy of the data. Although our findings align with existing literature, they are based on a specific population of medical and dental students, which constrains their generalizability to other groups, including those within the broader academic setting.

Furthermore, the cross-sectional design of the study aims to identify the prevalence of TMD in a specific population at a single point in time. As such, it does not allow for the establishment of causal relationships between the studied variables and the observed outcomes. Future studies should include standardized clinical evaluations and broaden the sample to include multiple universities. A potential response bias may also have occurred, as students with symptoms may have been more inclined to participate in a study on TMD.

## 5. Conclusions

Through this study, it was possible to investigate and confirm the high prevalence of temporomandibular disorder (TMD) symptoms among medical and dental students at a university in Brazil, thereby corroborating findings previously reported in the literature. Pain-related and joint-related TMD symptoms were significantly associated with high-frequency oral behaviors, as well as with moderate to severe levels of anxiety and depression. These results support the hypothesis that psychological factors and parafunctional oral habits are closely associated with the presence and severity of TMD in this academic population.

Recognizing and addressing these associations within university settings may facilitate the early identification of at-risk students and contribute to the development of preventive and supportive health strategies. Despite the methodological limitations of this study, the findings align with and reinforce existing evidence in the literature regarding the relationship between TMD and symptoms of anxiety and depression.
